# The “Ins and Outs” of Dynamic Magnetic Resonance Imaging for Female Pelvic Organ Prolapse

**DOI:** 10.1007/s00192-024-05935-9

**Published:** 2024-09-28

**Authors:** Eva K. Welch, Warren Ross, Katherine L. Dengler, Daniel D. Gruber, Shannon Lamb

**Affiliations:** 1 Department of Gynecologic Surgery & Obstetrics Urogynecology Division, Walter Reed National Military Medical Center, 8901 Wisconsin Avenue, Bethesda, MD 20889 USA; 2 Department of Radiology, United States Naval Hospital, Okinawa, Japan; 3 Division of Urogynecology, Sibley Memorial Hospital (Johns Hopkins Medicine), Washington, DC USA

**Keywords:** Anatomy, Dynamic MRI, Imaging modalities, Pelvic floor disorders, Prolapse, Rectal prolapse

## Abstract

**Introduction and Hypothesis:**

Concurrent pelvic organ and rectal prolapse have an incidence of 38%. Dynamic pelvic magnetic resonance imaging (MRI) is the modality of choice for workup. We discuss dynamic pelvic MRI indications, interpretation, and clinical application to pelvic floor disorders.

**Methods:**

The pubococcygeal line (PCL) extends from the pubic symphysis to the last coccygeal joint. The “H line” demonstrates the levator hiatus size, drawn from the inferior pubic symphysis to the posterior rectal wall at the anorectal junction. The “M line” represents vertical descent of the levator hiatus and extends perpendicularly from the PCL to the posterior aspect of the H line. With rectovaginal fascial defects, the small bowel, the peritoneum, and the sigmoid colon can prolapse. Posterior compartment abnormalities include rectocele, rectal prolapse, and descending perineal syndrome. Pelvic MRI can evaluate functional disorders such as anismus, where the anorectal angle is narrowed and associated with lack of pelvic floor descent and incomplete evacuation.

**Conclusions:**

Particularly for patients with concurrent urogynecological and colorectal complaints, previous pelvic reconstructive surgery, or when clinical symptomatology does not correlate with physical examination, dynamic pelvic MRI can impact management. It is critical for pelvic reconstructive surgeons to be familiar with this imaging modality to counsel patients and interpret radiographic findings.

**Supplementary Information:**

The online version contains supplementary material available at 10.1007/s00192-024-05935-9.

## Introduction

Concurrent pelvic organ prolapse and rectal prolapse have an incidence of at least 38% [1]. Risk factors include age, multiparity, vaginal deliveries, chronic constipation/straining, sigmoid colon redundancy, a deepened pouch of Douglas, and a posterior pelvic tilt [2]. Particularly with multi-compartmental prolapse, a multidisciplinary approach should be used in the workup and surgical management of these patients. Dynamic pelvic magnetic resonance imaging (MRI) has become the preferred modality of choice, particularly for posterior compartment disorders and defecatory disorders [3, 4]. Compared with older diagnostic methods such as fluoroscopic defecography, dynamic MRI circumvents the need for radiation, is not as invasive, and allows for simultaneous multiplanar evaluation of all pelvic compartments as well as soft tissues. Potential disadvantages include expense, which may limit widespread availability, and the patient’s nonphysiological supine position during the defecatory phase. As open-magnet systems become increasingly available, this can potentially improve patient comfort and ease when in sitting the evacuation position. Differences in the accuracy in diagnosis of pelvic floor and rectal pathology between the open- and closed-magnet systems, however, remain to be seen.

In 2017, the European Society of Urogenital Radiology and the European Society of Gastrointestinal and Abdominal Radiology published a joint recommendation regarding use of this modality [5]. The document provided guidance regarding patient indications, preparations, imaging protocol, imaging analysis/measurements/grading, and reporting in the hope of standardizing imaging techniques and reporting of dynamic MRI for pelvic floor disorders (PFDs). In 2021, the Pelvic Floor Disorders Consortium (PFDC), a multidisciplinary organization consisting of colorectal surgeons, urogynecologists, gastroenterologists, radiologists, and physiotherapists, developed a consensus statement regarding magnetic resonance defecography to generate guidance for all practitioners caring for patients with PFDs [6]. These recommendations further described techniques and templates that can then be tailored to patient indications and physician preferences/expertise. These documents are critical in advancing the understanding of this radiological technique. Our objectives are to build on the existing literature put forth by our professional societies and to provide an overarching review and discussion of dynamic pelvic MRI for PFDs in video format.

## Materials and Methods

This is an illustrative video with coincident interpretation of dynamic pelvic MR images and cine-loops.

## Results

As this can be a highly personal examination for the patient, it is imperative to clearly communicate with and gain feedback from the patient to minimize any embarrassment or discomfort. The patient is prepped by placing a medium such as ultrasound gel in the rectum as well as the vagina for opacification of the different compartments. Images are taken using a balanced steady-state free procession sequence in the mid-sagittal plane at rest, Valsalva or squeeze, straining, and evacuation.

Several lines for characterizing pelvic organ prolapse have been proposed. The pubococcygeal line (PCL) has the highest inter- and intra-observer reliability of the MRI-based reference points, perhaps as it is a line drawn between two easily identifiable bony points and is not influenced by pelvic tilt. It is obtained in the mid-sagittal plane at rest, with a line between the inferior border of the pubic symphysis to the last coccygeal joint. The PCL can be a helpful reference point particularly for cul-de-sac hernias such as peritoneoceles, sigmoidoceles, and enteroceles, and can also assist in differentiating between these hernias and posterior vaginal wall prolapse, which may appear to be similar on physical examination. The “hiatus” or “H line” demonstrates the antero-posterior width of the levator hiatus and is obtained on a midsagittal image with a line drawn from the inferior border of the pubic symphysis to the posterior wall of the rectum at the level of the anorectal junction. The “muscle” or “M line” represents the vertical descent of the levator hiatus and is drawn perpendicularly from the PCL to the posterior-most aspect of the H line. If a defect in the rectovaginal fascia is present, herniation of other tissues through the vagina, such as the small bowel, peritoneum, and sigmoid colon, can occur.

Posterior compartment abnormalities include rectocele and rectal prolapse. With the rectum prolapsing distal to the external anal sphincter and left untreated for a period as short as 2 years, permanent damage to the pudendal nerve can result, causing fecal incontinence even after surgical intervention. In contrast to rectal prolapse, descending perineal syndrome involves descent of the anorectal junction greater than 2.5 cm from the PCL. Pelvic MRI can also evaluate functional disorders such as paradoxical contraction of the puborectalis muscle or anismus. The anorectal angle is defined as the angle between the posterior border of the rectum and the central axis of the anal canal, is approximately 110–125° at rest, and normally increases by 15–20° with evacuation. With paradoxical contraction, the angle would not change or becomes more acute and is also associated with a lack of pelvic floor descent, leading to prolonged and incomplete evacuation.

## Conclusions

Dynamic pelvic MRI has become an imaging modality of choice for patients with complex prolapse. It is a useful adjunct to guide patient management, especially for patients presenting with concurrent urogynecological and colorectal complaints, those who have had previous pelvic reconstructive surgery, or when clinical symptomatology does not correlate with the physical examination. It is important to note, however, that radiographic findings should always correlate with clinical symptoms, as 25% of patients without symptoms may have measurements that exceed what is deemed normal [7]. As dynamic pelvic MRI gains popularity, it is critical for pelvic reconstructive surgeons to be familiar with this imaging modality to properly counsel patients regarding its process and accurately interpret radiographic findings.

## Supplementary Information

Below is the link to the electronic supplementary material.

Supplementary file1 (MP4 361523 KB)

## Declarations

### Disclaimer

The opinions expressed in this presentation are those of the authors and do not represent the Department of Health and Human Services or the Department of Defense.
